# A narrative review of the literature on illness uncertainty in hypermobile ehlers-danlos syndrome: implications for research and clinical practice

**DOI:** 10.1186/s12969-023-00908-6

**Published:** 2023-10-16

**Authors:** Estée C.H. Feldman, Kendra J. Homan, Sara E. Williams, Tracy V. Ting, Kenneth R. Goldschneider, Susmita Kashikar-Zuck

**Affiliations:** 1 Behavioral Medicine and Clinical Psychology, Cincinnati Children’s Hospital Medical Center, Cincinnati, OH USA; 2 Department of Pediatrics, University of Cincinnati College of Medicine, Cincinnati, OH USA; 3 Anesthesiology, Perioperative and Pain Medicine, Department of Anesthesia, Stanford University Medical School, Palo Alto, CA USA; 4 Division of Rheumatology, Department of Pediatrics, Cincinnati Children’s Hospital Medical Center, University of Cincinnati, Cincinnati, OH USA; 5 Pain Management Center, Department of Anesthesiology, Cincinnati Children’s Hospital Medical Center, Cincinnati, OH USA

**Keywords:** Hypermobility, Ehlers-Danlos syndrome, Illness uncertainty, Pain, Narrative review

## Abstract

**Background:**

Hypermobile Ehlers-Danlos syndrome (hEDS) is characterized by joint and skin laxity, and often accompanied by chronic pain, dysautonomia, increased distress and, functional limitations. The journey to accurate diagnosis is often prolonged due to unclear etiology of symptoms. This manuscript is a narrative review of the literature on illness uncertainty (IU) in hEDS, highlighting the unique facets of IU in this population, as compared to the broader chronic pain population (given symptom overlap between these two disease groups), that warrant additional investigation. Additionally, we considered the unique challenges associated with IU in the context of the developmental nuances of pediatric populations. Specifically, we aimed to (1) map the extant literature of the IU experience in chronic pain conditions broadly including the pediatric and adult research to identify key concepts related to IU and incorporate potential developmental considerations in IU; (2) delineate and describe the IU experience specifically in patients with hEDS, with the goal of identifying gaps in the literature based on aspects of presentation in hEDS that do and do not differ from the broader chronic pain population; and (3) elucidate the potential areas of adverse impact of IU in both general chronic pain populations, and those with hEDS specifically, to provide actionable areas for future research and clinical care of individuals with hEDS. Results of this review indicate that IU has been well-studied in chronic pain generally, but inadequately evaluated in hEDS specifically. Specific features of hEDS (complexity of the disorder, involvement of multiple bodily systems, contribution of organic pathology) may uniquely contribute to IU in this population. This review suggests that ambiguities surrounding the diagnosis of hEDS, symptom course, and treatment recommendations, along with misdiagnosis, perceived dismissal of symptoms, or attribution of symptoms to mental health concerns might increase risk for IU and related distress in patients.

**Conclusion:**

Findings from the present review suggest that distinct features of hEDS yield a set of driving factors for IU that may be somewhat different than those faced by patients with chronic pain or other medical conditions. The development of a validated measure of IU to appropriately assess this construct in patients with hEDS is a research priority. In the clinical setting, providers should be attentive to the potentially aversive diagnostic and treatment experiences reported by patients and attempt to provide clear explanations based on the extant knowledge of hEDS, and implement best-practice recommendations for multidisciplinary treatment.

## Background

Ehlers-Danlos syndrome (EDS) constitutes a heterogeneous group of connective tissue disorders with different types of the condition characterized by skin hyperextensibility, joint hypermobility, subluxation and dislocation of the joints, and general tissue and vasculature fragility [1, 2]. A subtype of EDS, hypermobile EDS (hEDS) accounts for greater than 90% of reported cases of this syndrome [3], and is frequently encountered in pediatric rheumatology settings. Unlike other types of EDS (e.g., classical, arthrochalasia, kyphoscoliotic, and vascular EDS; [4] no genetic testing or other diagnostic test is available for this subtype. Moreover, this subtype is distinguished from others by the increased frequency of generalized, chronic musculoskeletal pain, occurring in as many as 89% of patients with hypermobility syndrome or hEDS [5]. In fact, among patients with hEDS, joint pain presents frequently as the most common complaint [6, 7] with pain severity presenting as a leading cause of disability [8]. As hEDS continues to be classified as a rare disorder (and, therefore, is relatively less researched), and that the prevalent symptom is chronic pain, similarities and research may be drawn from the general chronic pain population in order to provide a theoretical background or basis for continued research within hEDS. Differences between hEDS and primary chronic pain disorders include potentially distinct underlying causes of pain in hEDS (structural abnormality in the form of joint hypermobility), additional co-morbid symptoms affecting multiple body systems and uniquely complex journeys within the healthcare system [9].

While no genes have been identified as causing hEDS, the impact of the disorder is lifelong. In fact, while initial presentation of symptoms occurs in childhood to early adolescence, on average, one study indicated that patients wait 22 years between the initial presentation of symptoms and receiving an appropriate diagnosis [10]. Therefore, education in the management of the condition must be rendered to both pediatric and adult providers, and consideration of the lifespan implications of the disorder is crucial. Early efforts to promote patient education is also important because although hEDS is vastly more prevalent than the other more rare and medically grave EDS subtypes, anecdotally, patients report engagement with online resources and other media outlets that warn of potential dire and life-threatening symptoms. The fact that the diagnosis of hEDS is primarily made based on physical exam and self-report of symptoms (e.g., joint hypermobility, subluxation, pain) may be unsatisfying to families who may feel the need for a more comprehensive diagnostic work-up to identify or rule out other more concerning EDS subtypes; in fact, in a review [11] of qualitative studies assessing diagnostic delays in adults with hEDS, a lack of confirmatory test was cited as a key perceived barrier by patients to receiving appropriate diagnosis. Additional cited diagnostic barriers included the range of symptoms experienced, provider attitudes (i.e., attributing symptoms to mental illness), suggesting that while hEDS is generally considered a less severe form of EDS, patients may not be easily reassured because of the uncertainty surrounding the hEDS diagnosis and its proper medical management.

This perceived loss of control regarding one’s illness and its treatment is defined as illness uncertainty (IU), and research has shown that IU is often associated with maladaptive coping, higher psychological distress, and reduced quality of life [12]. More specifically, among patients with fibromyalgia, IU has been associated with depression, anxiety, heightened negative affect and reduced positive affect, maladaptive (passive and avoidant) coping styles and diminished coping efficacy, and limited adjustment to acute stress and pain [13, 14]. Among those with rheumatoid arthritis, IU has been associated with perception of increased illness severity, poorer health-related quality of life, and diminished utilization of self-help behaviors [15]. In brief, other studied disease populations include diabetes mellitus [16], breast and gynecological cancers [17–19], postpolio syndrome [20], Parkinson’s disease [21], and multiple sclerosis [22]. Findings generally indicate that higher levels of IU are associated with more frequent “stress-related (i.e., not attributed to disease process) hospital visits [18], diminished hope [17, 23], greater illness intrusiveness [21], poorer spiritual well-being [16], and poorer mental health (specifically more anger, tension, anxiety and depressive symptoms; [16, 22, 24, 25].

While findings are robust in other disease populations, research within adults, and to an even greater extent, pediatric hEDS is sparse due in part to the lack of validated measure of this construct within this population. In fact, studies in adults with hEDS to date that have attempted to study this construct utilizing well-validated, pre-existing measures (i.e., the Mishel Uncertainty in Illness Scale; [26] have been unable to rely on findings from the measure due to poor internal reliability of the Unpredictability Subscale (α = 0.5947; [27]. To begin to address the gap in the literature and guide the development of a measure of IU with appropriate construct validity, attention must first be devoted to understanding through qualitative research and review how this construct presents uniquely within this population. Moreover, while some limited literature documents the occurrence of certain facets of uncertainty within adult hEDS populations [11, 28], the construct has been studied to an even lesser extent among pediatric hEDS populations, who may present with a different IU experience given developmental differences, and a relatively abridged journey in managing the illness (as compared to adults with hEDS who have managed it their entire lives). Therefore, consideration of the unique presentation and ramifications of IU in pediatric hEDS is crucial in order to provide a framework to guide the development of future clinical early intervention, given preliminary evidence of adverse effects of IU in hEDS more broadly [5, 6].

Therefore, the present narrative review was guided by the following aims:

1. To map the extant literature of the IU experience in chronic pain conditions broadly including the pediatric and adult literature to identify key concepts related to IU and incorporate potential developmental considerations in IU;
2. To delineate and describe the IU experience specifically in patients with hEDS, with the goal of identifying gaps in the literature based on aspects of presentation in hEDS that do and do not differ from the broader chronic pain population;
3. To elucidate the potential areas of adverse impact of IU in both general chronic pain populations, and those with hEDS specifically, to provide actionable items for future research efforts and implications for clinical care of individuals with hEDS.

## Methods

The present narrative review was conducted to summarize current understanding of IU in pediatric and adult pain populations generally and examine the state of the literature in hEDS specifically to highlight domains of IU unique to this population. Following the guidance on how to conduct a narrative review utilizing a “best evidence- synthesis” [29], the present methodology section highlights steps taken to collect and unbiasedly synthesize information for the reader. Inclusion and exclusion criteria were decided upon through consultation with the senior author (SKZ). Both qualitative and quantitative studies, as well as review articles, were included in the present search to maximize findings. Similarly, the grey literature, when available, was included. This broad approach allowed for the identification of all relevant literature, regardless of study design [30]. Only material available in English was included, due to time and cost associated with appropriate translation. To ensure consistency of search items and engines utilized, the literature search was conducted solely by the first author (EF). The following key terms were searched, based on the decided criteria: terms related to IU (illness/diagnostic/treatment uncertainty AND/OR clarity, ambiguity, complexity, symptom misattribution, misdiagnosis) in (pediatric AND/OR) adult chronic pain AND (pediatric AND/OR adult) hypermobile Ehlers-Danlos Syndrome (AND joint hypermobility syndrome, benign joint hypermobility syndrome, hypermobility syndrome, hypermobility spectrum disorder). These terms were searched in each unique combination in Google Scholar and PubMed. Duplicate articles were not counted twice. All manuscripts reviewed utilizing the above-mentioned search terms were included, except for those in which there was an identified medical diagnosis which accounted for the chronic pain (e.g., pain due to cancer or sickle cell disease). Articles included were also reviewed for appropriateness for inclusion (based on the same search terms) of materials referenced therein.

Themes were determined utilizing the data charting method outlined by Arksey and O’Malley [30] wherein data is synthesized and interpreted by sorting material according to key issues and themes. They encourage a narrative review utilizing a descriptive-analytical approach, characterized by applying a common analytic framework to all works reviewed to allow for the collection of standardized information in each study [30]. To this end, data was collected from each reviewed manuscript on the broad domains of (1) sources or causes of IU and (2) adverse impacts of IU. As pervasive themes within these broader domains became apparent, they were categorized further within these domains and findings within said themes summarized across studies. Based on these findings, relevance for clinical intervention and further research are discussed.

## Results

The literature review resulted in the utilization of 35 articles, 7 in chronic pain generally, and 16 in EDS generally, hEDS specifically, or other hypermobile populations. Twelve studies reported on non-clinical populations. Of this total, 7 reported on pediatric populations (with or without report from parents as well), 18 on adult populations, and 5 used a lifespan sample. Five studies did not report on age (i.e., commentaries, framework proposals that did not report on specific samples). Five qualitative studies, 11 quantitative, 2 mixed method, 5 experimental, and 6 review studies were included. Six studies were otherwise categorized (e.g., case studies, position papers, commentaries). Please see Table 1 for a summary of each study’s findings.

The initial broader domain of sources or causes of IU was divided into several themes, some of which were shared between the broader chronic pain population and, in hEDS specifically, and others of which were unique to each respective disease group.

### Causes of IU in Chronic Pain

#### Diagnostic uncertainty: reckoning with a diagnosis of exclusion

Individuals with pain syndromes may be particularly likely to experience high levels of IU with respect to etiology of their pain diagnosis and their understanding of the rationale for treatment. Findings from the present review suggest that a significant proportion of both parents and patients at a pediatric pain clinic in the USA had diagnostic uncertainty [31]. Many times, such patients arrive at such a clinic to be given a chronic pain diagnosis after being told all diagnostic maneuvers are normal. Thus, a pain diagnosis often appears to patients as one of unclear etiology and as a “diagnosis of exclusion” after other medical conditions have been ruled out. This diagnostic process proves challenging for many parents of children with chronic pain, who report struggling to accept a diagnosis for their child based on lack of organic findings [32]. As a result, they are likely to seek further medical or organic explanation for their child’s pain [32]. This uncertainty may prove especially distressing for youth with pain, given the concrete thinking characteristic of childhood and early adolescent years [33]. In one study [31], nearly half of children and over one third of parents surveyed reported believing that there was a cause for the child’s pain that was not yet discovered. This belief, in turn, was associated with greater avoidance of physical activities of these pediatric patients, lower pain acceptance, and decreased adaptation to and engagement with developmentally appropriate daily activities [31].

### Treatment uncertainty: a perceived lack of options and dismissing message

Uncertainty regarding treatment course is similarly complex. Findings from the present review reveal that recommended interventions are often non-pharmacologic, which may elicit feelings of being dismissed by the medical community among youth with pain [32]. Among adults with chronic pain, further skepticism can arise from the lack of knowledge about the effectiveness of non-pharmacologic interventions, presenting an additional barrier to engagement in evidence-based treatments for chronic pain [34].

### Adverse impacts of IU in Chronic Pain

Uncertainty about diagnosis and treatment can have adverse implications for the pain experience and outcomes. IU has been found to be associated with heightened pain sensitivity, intensity, attentional focus, and greater pain interference in both clinical and research settings [35–37] and in both pediatric [33] and adult populations [35–37]. Interestingly, brain areas responsible for the affective components of pain, attention to pain, and expectation of pain (e.g., anterior cingulate cortex, parietal operculum, posterior insula) have been found to be significantly more engaged in research participants who were presented with non-painful stimulation in an uncertain context (i.e., unaware as to the type of stimuli they would be receiving, painful or non-painful) as compared to matched controls who knew they would receive only non-painful stimuli [38].Results suggest that uncertainty itself may amplify the neural processing and subjective unpleasantness of even non painful stimuli [38].

In addition to impacting neural underpinnings of pain processing, IU has a considerable impact on cognitive processes, specifically with regard to appraisals of pain-related threats. Longitudinally, greater IU was found to predict higher pain interference via promotion of pain catastrophizing and increased fear of pain in youth with chronic pain [35]. Similarly, greater parent IU predicted greater parent pain catastrophizing, in turn promoting increased protectiveness, greater youth fear of pain, and increased pain interference. Such findings highlight the family-system wide impact of IU on aversive pain experiences. Among adults with chronic pain, IU has also been associated with broader negative psychosocial effects, including higher rates of psychopathology (depression and anxiety [13] and maladaptive coping (avoidant and passive coping; [13], lower levels of coping efficacy; [14].

### Unique facets of causes of IU in hEDS

While hEDS is similar to chronic pain disorders in the frequent experience of widespread pain and the experience of diagnostic uncertainty, a nuanced review of the literature suggests different lived disease-related experiences driving this uncertainty (i.e., uncertainty due to confusion secondary to diagnosis of hEDS vs. joint hypermobility syndrome as well as subtyping of EDS, potential misdiagnoses and misattributions of symptoms by health care professionals, rather than being given a “diagnosis of exclusion” as is seen in broader chronic pain populations) and 2). Moreover, due to the proposed pathophysiology underlying hEDS, quite uniquely, individuals experience overlapping symptoms and altered interoceptive cues (e.g., tachycardia, tingling, lightheadedness, nausea) due to the symptoms of hEDS itself, as well as frequently encountered comorbidities, such as mast cell activation syndrome (MCAS) and postural orthostatic tachycardia syndrome (POTS), the symptoms of which inherently promote uncertainty and confusion, and the pathophysiology of which are largely unknown and criteria based on subjective report [39].

### Diagnostic uncertainty: cut-off confusion, hEDS versus JHS versus HSD, and misdiagnosis and misattribution of symptoms

A diagnosis of hEDS is made via clinical criteria that include the Beighton Scale and a series of objective features and subjective complaints [40]. Despite efforts made to ensure standardization of diagnosis, both clinical judgment and evolving diagnostic cutoffs preclude provision of a consistent, clear diagnosis.

Current findings highlight the inconsistency of standards for diagnosis reported in the available literature, which reflect those used in clinical practice. For adults with hEDS, recommendations for diagnostic cutoffs have varied over time. While a score of 3/9 was initially indicated [41–43], more recently, research and clinical practice indicates a score of 4 [44], or 5/9 is appropriate [45]. Among children, greater stringency is recommended although exact diagnostic cut-off is similarly debated: some suggest a score of 6/9 indicates the generalized joint laxity characteristic of hEDS [45], while others suggest a score of 7/9 is needed to mitigate the risk of “false positive” diagnoses of hEDS [46] given that children have increased mobility and higher levels of subcutaneous fat [47, 48]. Moreover, since additional factors (e.g., age, sex, ethnicity; [49] are known to influence Beighton scores, specific age and sex cut-offs to minimize “false positive” diagnoses are indicated, yet have not been implemented in clinical settings, or developed by research.

Further confusing matters for patients is the diagnosis of joint hypermobility syndrome, or its proposed replacement term “hypermobility spectrum disorder(s)”, which refers to patients who have many of the findings and symptoms of hEDS, but who do not meet full criteria per the 2017 recommendations [40, 50].

Other challenges in early and accurate diagnosis can come from misattributions from health care professionals including (1) concerns of child abuse/endangerment [51] due to frequent injuries, subluxations and emergency department visits, (2) diagnoses of conversion disorder (now referred to as functional neurological symptom disorder [52] or (3) factitious disorder or (4) broader dismissal of symptoms as caused by mental health concerns (e.g., anxiety or depression; [53]. Of note, while anxiety and depression are frequently comorbid with chronic pain independent of hEDS [53], symptoms of hEDS may overlap with somatic symptoms of anxiety and depression and, therefore, be dismissed instead of concurrently identified and treated. For example, dysautonomia, a frequently noted comorbidity in hEDS, is characterized by a broad set of symptoms (e.g., heart rate and blood pressure fluctuations) which may mimic anxiety and/or panic attacks. Similarly, poor sleep and/or high levels of fatigue (with 86% of patients endorsing chronic asthenia/fatigue, [54]) patients with hEDS could be attributed to depression.

### Overlapping symptomatology and alteration of interoceptive cues

While symptoms of hEDS should not be dismissed as somatic psychopathology symptoms, findings from the research surveyed suggests there are shared mechanisms underlying anxiety and symptoms of hEDS. This shared mechanism explains the increased risk of clinically significant anxiety for individuals with hEDS, which further predisposes them to the experience of IU, and adverse IU effects. Greater hypermobility scores have been associated both with increased activation of affective centers of the brain [55], as well as increased structural volumes of these same regions (i.e., larger bilateral amygdala in patients with hypermobility; [56]. These changes are proposed to be linked to heightened interoceptive sensitivity [55, 57], which may be tied to the high frequency of anxiety seen in individuals with hEDS [58]. Anxiety has been tied to increased somatosensory amplification, catastrophizing, reduced function, poor sleep [59], and somatic complaints in hEDS, including kinesiophobia [54] and hyperalgesia [60]. This is consistent with existing theories of emotion, which posit that we come to recognize our emotional state by first identifying our internal physical sensations (i.e., James-Lang Theory of Emotion; [61, 62]. Therefore, according to such a theory, in the body of an individual with hEDS, who may regularly experience a racing or pounding heart, sweating, and pain, a message may be communicated of lack of safety, threat, or fear in order to elicit protective responses from the brain and body. Given the frequency with which these symptoms occur, this may promote a state of sustained anxiety or worry about one’s physical safety that is exacerbated by uncertainty in one’s diagnosis (i.e., fearing something more severe or immediately threatening) and treatment plan (i.e., worrying that serious symptoms are not being readily addressed), or even in one’s provider (i.e., worrying that the provider is “missing something” in their work-up, or diagnosis).

### Adverse impact of IU in hEDS

Findings from the present review suggest that the adverse impact of IU in hEDS often reflect the unique facets of IU experienced by this population, as compared to the broader chronic pain population. For example, in consideration of the unique facets of diagnostic uncertainty, the lack of clarity around diagnostic cut-offs and lack of appropriate terminology utilization can influence a provider’s ability to provide a clear diagnosis with some health care providers indicating a reluctance to diagnose children with hEDS [63]. As a result, time to appropriate diagnosis and initiation of treatment including symptom management, and functional assistive devices (if appropriate) may be delayed [64, 65]. Similarly, related to the frequent reports of misattribution of symptoms and misdiagnosis, findings from qualitative interviews reviewed indicate that individuals with EDS can feel dismissed, psychologically labelled or suspected of family violence [66]. Such perceptions may promote distrust in the medical team and undermine their trust in and adherence to recommended treatments [66]. Finally, the predisposition to physiologic symptoms of anxiety, paired with the described intense interception [55] and sensory amplification [59] predispose individuals with hEDS to the accompanying *cognitive* manifestations of anxiety, due to the role of perception and interpretation of physiological excitation in anxiety disorders [67–69]. High rates of anxiety disorders are noted in this population - generalized anxiety [59, 70, 71] and panic disorders [72] are noted in about 60–70% of hEDS patients, as well as an elevated risk (as high as 6 times greater than the general population, ~ 11%) of obsessive-compulsive personality disorder [73–75]. Such high rates of distress may predispose individuals with hEDS to poor adaptation in the face of the extensive IU they face.

## Discussion

The present review summarizes the experience of IU in chronic pain populations generally, and within hEDS specifically. Findings from the present review suggest that while a primary symptom (pain) is shared between the two disease groups, distinct features of hEDS yield either different, additional, facets of uncertainty entirely or result in different driving forces underlying shared facets of uncertainty (i.e., diagnostic uncertainty). This finding suggests that hypermobility itself may, consistent with associations with previously documented structural and functional brain changes [55, 56] present as a risk factor for uncertainty, above and beyond what can be explained by the experience of the pain it espouses. Per a qualitative study of adults with EDS [28], causes of this uncertainty uniquely associated with hypermobility may include a a “fear of the unknown”, characterized by a fear of future decline, a lack of reliable information about their diagnosis, and effects on pregnancy/heritability.

This uncertainty may be due to symptoms associated with hypermobility itself, the fact that the diagnosis is based on clinical criteria (without genetic or other objective testing), and is frequently associated with a number of other diagnoses (e.g., POTS, MCAS; [76, 77], GI symptoms and disorders; [78]) [79], which may in turn have unclear and shifting diagnostic criteria, further magnifying and maintaining uncertainty. This is of specific concern, given the previously discussed adverse impacts of IU in this population, including increased time to appropriate diagnosis and related treatment [64, 65], perception of judgment from medical providers [66] which may further undermine trust in and adherence to treatment recommendations [66] and high rates of anxious psychopathology [59, 70–75].

## Conclusions

Despite the notable impact of IU, it has been minimally studied in part due to the lack of validated measure for pediatric or adult hEDS. The lack of construct validity of the standard, existing measure of this construct calls for the creation of a measure which accurately represents the lived experience of uncertainty within this distinct disease population. Given that symptoms most frequently first present in childhood, measure development is encouraged to focus on this subset of patients to foster early identification of IU, and ideally, early intervention to promote adjustment.

Clinically, the significant uncertainty characteristic of the diagnostic, prognostic, and treatment course of hEDS holds implications for the rheumatology clinics these patients present to. Due to their long journeys to diagnosis, perception of dismissal from health-care providers, and misattribution of symptoms, patients with hEDS have often experienced delays in access to appropriate care and, as a result, may be experiencing diminished psychosocial well-being [28, 66, 80–83]. These adverse effects may be amplified in the pediatric setting, due to additional healthcare provider reluctance to diagnose children [63], the exacerbation of symptoms due to hormone fluctuation in puberty which may magnify disability and distress [84] and the lack of guidelines for treatment of hEDS in pediatric populations [64]. To address the aforementioned domains of distress and foster positive patient-provider relationships [85] families of children with hEDS have identified addressing diagnostic and treatment uncertainty as valuable [85]. Therefore, clinicians are encouraged to 1) recognize and validate any challenges in reaching diagnosis, including consideration of frequently co-occurring diagnoses (e.g., POTS, MCAS; [76, 77]; GI conditions including abnormal colonic transit, gastric emptying, esophageal manometry, and/or pathologic acid reflux; [78], and irritable bowel syndrome/ IBS [79]) assess for negative experiences with healthcare providers which may influence the current therapeutic relationship and undermine treatment engagement, and 3) collaboratively create a clear treatment plan with the patient and their families. Given adverse outcomes associated with IU, clinicians are encouraged to utilize clinical interviews to assess domains of uncertainty (diagnostic, prognostic, treatment), as well as perception of prior dismissal from the healthcare system to establish rapport and collaboration.

**Table 1:** Overview of the reviewed sources

Author, Year	Population	Diagnosis(es)	Study Design	Measures	Aim(s) of the Study	Key Findings
Acasuso Diaz et al., 1993	675 teenage soldiers (male only)	Non-clinical healthy sample	Quantitative	Degree of joint hypermobility assessed by 5 criteria	To determine the prevalence of hypermobility (cutoff of 2–3/5 and 4–5/5) among young male soldiers	25.5% of soldiers met 2 or 3 criteria and 7.5% met 4 or 5 criteria; Injury was significantly more frequent than in soldiers with normal joint mobility
Al-Rawi, Al-Aszawi, & Al-Chalabi, 1985	University sample of 1774 young adults	Non-clinical healthy sample	Quantitative	Hypermobility assessed via Beighton Scale; Height/weight	To determine the prevalence of hypermobility (cutoff of 4/9) among university students	Prevalence of hypermobility was high (males = 25.4%; females = 38.5%)
Baeza-Velasco et al., 2011	University sample of 365 young adults	Non-clinical healthy sample	Cross-sectional quantitative	Hypermobility assessed via Beighton Scale; Self-report measures	To determine the frequency of hypermobility among university students; To explore the relationship between hypermobility, somatosensory amplification Scale, depression, and anxiety	Somatosensory amplification was higher in students with hypermobility independent of gender; Depression and anxiety were higher in female students with hypermobility; social anxiety was higher in male students with hypermobility
Bair et al., 2003	Review of studies that included adults only	Chronic pain	Literature review	Search: Depression or depressive disorders and pain	To determine the prevalence of depression and pain and the effects of comorbidity on diagnosis, clinical outcomes, and treatment	65% of patients with depression experienced pain and between 5–85% of patients with pain experienced depression – rates that are higher than when the conditions are examined individually; Both pain and depression were negatively associated with poor pain outcomes and worse prognosis
Barnum, 2014	Pediatric patient	hEDS	Case study	N/A	To discuss the impact of an inaccurate diagnosis of conversion disorder^a^ in a pediatric patient with hEDS	Diagnosis of conversion disorder can undermine patients’ trust and create defensiveness that may interfere with acquisition of appropriate diagnosis, and related treatment.
Becker et al., 2017	26 adult patients and 26 practitioners	Chronic pain	Qualitative	Semi-structured interview	To identify factors related to whether one utilizes evidence-based non-pharmacologic pain treatment	Patient themes: Barriers – high cost, transportation difficulties, low motivation; Facilitators – greater availability of treatment, team-based treatment with follow-up; Practitioner themes: Barriers – inability to promote non-pharmacologic treatment after opioids, patient skepticism; Facilitators – consistent treatment philosophy, increased patient knowledge about non-pharmacologic treatment
Berglund, Anne-Cathrine, Randers, 2010	22 adults	EDS	Qualitative	Study-specific questionnaire	To describe health-care encounters patients with EDS experienced in which their dignity was not upheld and the long-term consequences associated with these encounters	Themes: Ignored/belittled. assigned psychological explanations, treated as an object, personal space invaded, questioned about family violence; Consequences of these encounters included mistrust and negatively impacting on health
Bulbena et al., 2015	Review of studies that included adults	hEDS	Literature review	N/A	To summarize research concerning the relationship between hypermobility and anxiety disorders	The relationship between hypermobility and anxiety disorders have been well established; Common mechanisms that are involved in include genetics, autonomic nervous system dysfunctions, and interoceptive/exteroceptive processes
Castori, 2015	N/A	EDS including hEDS	Editorial/ commentary	N/A	To aid in practitioners in the differentiation of trauma due to EDS versus abuse	EDS should be considered in the differential diagnosis of children with a suspect of non-accidental injury such as skin lacerations, bruising, dislocations
Castori et al., 2013	Review of studies that included children and adults	EDS including hEDS	Reinterpretation of the literature	Search: joint laxity/joint instability/EDSand pain, fatigue, or headache	To re-interpret the published literature (based on the authors’ multidisciplinary clinical experience) on pain, fatigue, and headache in EDS based on authors’ multidisciplinary clinical experience	Pathogenic mechanisms of pain, fatigue, and headache in hEDS are offered through comparisons with other functional somatic syndromes
Castori et al., 2017	N/A	hEDS	Editorial/ commentary	N/A	To propose a framework for the classification for joint hypermobility-related disorders	A continuous spectrum ranging from symptomatic joint hypermobility to hypermobility spectrum disorders to hEDS should be used; This spectrum supports the dynamic nature of condition.
Celletti, et al., 2013	42 adult patients	hEDS	Cross-sectional quantitative	Self-report measures	To investigate the impact of kinesiophobia in hEDS and the relationship with pain, fatigue, and QoL	Kinesiophobia is common in hEDS; severity of kinesiophobia was related to severity of fatigue and, generally, related to severity of pain but not to QoL, or to intensity of pain or fatigue
Clinch et al., 2011	Population-based cohort of 6,022 children	Non-clinical healthy sample	Quantitative	Hypermobility assessed via Beighton Scale; Height/weight; Assessment of physical activity, puberty, and SES	To determine the point prevalence and pattern of hypermobility (cutoff of 4/9) in children from a population-based cohort	Prevalence of hypermobility was high in children (girls = 27.5%; boys = 10.6%) suggesting that the cutoff of 4/9 is too low for this population
De Baets et al., 2017	10 adult females who have had at least 2 children	hEDS	Qualitative	Semi-structured interview	To explore the lived experiences of women with hEDS regarding diagnosis, influence on daily life, and motherhood	Themes: Relief in receiving diagnosis/support to become a mother, hEDS emotionally related distress impact on social/physical behavior, adjustment of everyday activities, differing mother/child expectations, importance of supportive social/physical environment, and child decreases illness focus of mother
Eccles et al., 2012	72 adults	Non-clinical healthy sample	Experimental	Hypermobility assessed via Beighton Scale; Self-report measures; fMRI	To examine the relationship between regional cerebral grey matter and hypermobility (cutoff of at least 1/9) using fMRI	Structural differences in the key emotion-processing brain regions and decreased volume within other regions implicated in emotional arousal and attention were found in the group with hypermobility as compared to those without.
Engelbert et al., 2017	N/A	hEDS	Practice guideline	N/A	To provide education as to the role of PT in the assessment and management of hEDS in both pediatric and adult populations:	Descried the following factors as key for management of pain in hEDS: proprioception, muscle strength and balance; joint instability; extra-articular factors; psychological symptoms; motor development, gait pattern, physical fitness; and participation in hobbies, sports, and social activities
Grahame, 2017	N/A	EDS including hEDS	Editorial/ commentary	N/A	To correct two misconceptions about hEDS and the resulting hesitancy to diagnose hEDS in pediatric populations	Two misconceptions identified are that symptomatic joint hypermobility occurs in otherwise healthy individuals and the dismissal of an underlying connective tissue disorder; Encouragement provided to consider early diagnosis and intervention
Johnson, Zautra, & Davis, 2006	51 adults (female only)	Fibromyalgia	Quantitative and qualitative	Self-report measures; Weekly semi-structured interview for 10–12 weeks	To examine the relationship between IU in pain coping focusing on weeks with greater pain intensity	For participants with high IU, pain severity predicted increases in coping difficulty; Coping difficulty was associated with lower coping efficacy
Juul-Kristensen et al., 2017	Review of studies that included children and adults	JHS/hEDS	Systematic review	Search one: combinations of joint, laxity, hypermobility, instability, general and evaluation, rate, questionnaire, test, examine, scale, diagnose, assess, observe, measure; Search two: added psychometrics, clinometric, reproducibility, reliability, repeatability, responsiveness, sensitivity, specificity, validity, diagnosis, feasibility	To complete a systematic review of the clinical assessment methods for classifying generalized joint hypermobility	6 measures of hypermobility were identified with most studies using the Beighton Scale; inter-rater reliability was acceptable, however, more research on the validity is needed; when using the Beighton Scale, a cutoff of 5/9 criteria for adults and 6/9 for children is used provided uniformity of testing procedures
Kennedy et al., 2022	Review of studies that included children and adults	EDS including hEDS	Systematic review	Search: Ehlers-Danlos syndrome and psychology or mental disorder	To complete a systematic review of the psychiatric disorders in the EDS population	63.2% of patients with EDS were diagnosed with a language disorder, 52.4% with attention-deficit/hyperactivity disorder, 51.2% with anxiety, 42.4% with a learning disability and 30.2% depression
Klemp & Learmonth, 1984	47 adult ballet dancers and age-/sex-matched controls	Non-clinical healthy sample	Longitudinal (10 years) quantitative	Hypermobility assessed via Beighton Scale; Rate of injury	To determine the prevalence of hypermobility (cutoff of 4/9) among ballet dancers and frequency of injury	Ballet dancers were not found to be more hypermobile and did not sustain more injuries as compared to age-/sex-matched controls
Kohn & Chang, 2020	Review of studies that included children and adults	hEDS, POTS, and MCAS	Literature review	Search one: Various combinations of hEDS, POTS, and MCAS; Search two: Various combinations of all forms of EDS, POTS, and MCAS	To review the comorbidity between hEDS, POTS, and MCAS	An evidence-based pathophysiologic relationship between hEDS and POTS or MCAS does not exist and studies describing a relationship are biased or based on outdated criteria
Malek, Reinhold, & Pearcem, 2021	Review of studies that included adults only	hEDS	Literature review	Search one: Beighton Score and validity, correlation, or reliability; Search two: Expanded to include various joints	To review the validity of the Beighton Score as a diagnostic tool for hypermobility	As the Beighton Score does not accurately represent the diagnosis definition of and should not be used as a direct indicator of generalized joint hypermobility
Malfait et al., 2017	N/A	hEDS	Position paper	N/A	To propose a revised hEDS classification system be used for clinical and research purposes	Outlined clinical criteria for hEDS to allow for greater distinction from other heritable connective tissue disorders
Mallorqui-Bague et al., 2014	36 adults	Non-clinical healthy sample	Experimental	Hypermobility assessed via Beighton Scale; Self-report measures; Interoceptive sensitivity assessed via heartbeat detection task; fMRI	To examine the relationship between anxiety, interoceptive sensitivity, and hypermobility (cutoff of 5/9 for women and 4/9 ≥ 4 for men) using fMRI	Anxiety and hypermobility are related, and the relationship is mediated by interoceptive sensitivity; Participants who were hypermobile displayed heightened neural reactivity to brain regions implicated in anxious feeling states
Neville et al., 2019	20 pediatric patients and their parents	Chronic pain	Qualitative	Semi-structured interview	To explore the lived experience of IU in pediatric patients their parents	Themes included IU associated with the function/meaning of the diagnosis, worry surrounding something missing, search for an alternative diagnosis, and mistrust in the medical system
Neville, et al., 2021	152 children and their parents	Chronic pain	Longitudinal (3 months) quantitative	Self-report measures	To examine the association between IU and the Interpersonal Fear Avoidance Model of Pain	Parent and child IU were identified as risk factors in the maintenance of pediatric chronic pain at 3 months through parent and child pain catastrophizing, parent protectiveness, and youth fear of pain.
Palmer et al., 2016	25 adult patients and 14 practitioners	hEDS^2^	Qualitative	Focus groups (conducted separately for patients and practitioners)	To explore patient and practitioner views on PT in the treatment of hEDS	Themes included PT is ineffective for acute joint problems and if diagnosis is delayed, and effective PT included therapist who is familiar with hEDS, patient led, flexible, and takes a long-term approach
Ploghaus et al., 2001	8 adults (male only)	Non-clinical healthy sample	Experimental	Self-reported pain intensity, Event-related fMRI	To examine the neural mechanisms of induced anxiety and nociceptive stimulation perception of pain via event-related fMRI	Anxiety-induced hyperalgesia is associated with increased activation of portions of the hippocampal formation (consistent with Gray-McNaughton Theory). Authors suggest that interventions which modulate hippocampal activation may be valuable for management of both procedural and chronic pain.
Reich et al., 2006	51 adults	Fibromyalgia	Quantitative and qualitative	Self-report measures; Weekly semi-structured interview for 10–12 weeks	To examine relationship between IU and depression, anxiety, affect, and coping styles	IU was associated with anxiety, negative affect, avoidant coping, and passive coping and, during stress, IU was found to be a risk factor for negative affect
Rhudy & Meagher, 2000	University sample of 60 young adults	Non-clinical healthy sample	Experimental	Exposure to electric shock was used to induce fear, whereas anticipation of shock (without exposure) was used to induce anxiety. Exposure to electric shock was used to induce fear; Anticipation of shock (without exposure) was used to induce anxiety	To examine the effects of experimentally induced fear and anxiety pain thresholds using fMRI	Experimentally induced anxiety increased pain reactivity while experimentally induced fear resulted in decreased pain reactivity
Sawamoto et al., 2000	10 adults (male only)	Non-clinical healthy sample	Experimental	Self-reported pain intensity and pain unpleasantness; Event-related fMRI	To examine whether the expectation of pain amplifies brain responses to somatosensory stimulation in areas of the brain that regulates behavioral reaction to pain using fMRI	Uncertain expectation of pain amplifies areas of the brain (anterior cingulate cortex, parietal operculum, and posterior insula) which regulates behavioral reaction to pain
Singh et al., 2017	1000 children and adults	Non-clinical healthy sample	Cross-sectional quantitative	Hypermobility assessed via Beighton Scale	To evaluate distribution of Beighton scores (cutoff of 4/9) in a healthy population	Beighton score of 4/9 yielded a high false positive rate of 60% suggesting overestimation of prevalence with this cutoff; Cutoffs should be varied across the life span with age-/sex-specific values cutoffs
Smits-Engelsman, Klerks, & Kirby, 2011	551 elementary school-aged children	Non-clinical healthy sample	Quantitative	Hypermobility assessed via Beighton Scale	To determine the prevalence of hypermobility and the validity of the Beighton scale (cutoff of 5/9) in elementary school aged children	Prevalence of hypermobility was high (35.6%; no sex differences) suggesting that a stricter cutoff score be used; Complaints of join pain and pain after exercise were not significantly different between children with more or less hypermobility
Tanna et al., 2020	91 pediatric patients and 126 of their parents	Varied pain locations/diagnoses including hEDS	Quantitative	Self-report measures	To examine the prevalence and familial concordance of IU and the relationship between parent and child IU with several parent and child psychological factors	Parent IU was associate with higher avoidance of pain-related activities and lower pain acceptance in their children; Parent and child IU was related to the child’s functioning

*Note*. EDS: Ehlers–Danlos syndrome; IU: Illness uncertainty; HEDS: hypermobile Ehlers-Danlos syndrome; MCAS: Mast cell activation syndrome; POTS: Postural orthostatic tachycardia syndrome; PT: Physical therapy: QoL: Quality of life

^a^Conversion disorder is now known as functional neurological disorder/ functional neurological symptom disorder (FND/FNSD)

## Data Availability

Not applicable.

## Abbreviations

hEDS: Hypermobile Ehlers—Danlos Syndrome
IU: Illness uncertainty
POTS: Postural orthostatic tachycardia syndrome
MCAS: Mast cell activation syndrome
